# The evolution of the cancer formulary review in Canada: Can centralization improve the use of economic evaluation?

**DOI:** 10.1002/hpm.2372

**Published:** 2016-07-29

**Authors:** W. Dominika Wranik, Liesl Gambold, Natasha Hanson, Adrian Levy

**Affiliations:** ^1^ School of Public AdministrationDalhousie UniversityHalifaxNova ScotiaCanada; ^2^ Department of Sociology and Social AnthropologyDalhousie UniversityHalifaxNova ScotiaCanada; ^3^ Saint John Regional HospitalSaint JohnNew BrunswickCanada; ^4^ Department of Community Health and EpidemiologyDalhousie UniversityHalifaxNova ScotiaCanada

**Keywords:** drug reimbursement, formulary decisions, cancer drugs, multiple decision criteria, multidisciplinary review committee

## Abstract

Public reimbursement of drugs is a costly proposition for health care systems. Decisions to add drugs to the public formulary are often guided by review processes and committees. The evolution of the formulary review process in Canada's publicly funded health system is characterized by increased centralization and systematization. In the past, the review of evidence and recommendation was conducted at the regional level, but was replaced with the pan‐Canadian Oncology Drug Review in 2011. We assess the extent to which centralization and systematization of the review process have responded to past challenges, focusing on the use of economic evaluation in the process. Past challenges with economic evaluation experienced by regionalized review committees were identified from literature and qualitative data collected in the province of Nova Scotia. We categorize these using a typology with a macro‐, meso, and micro‐level hierarchy, which provides a useful framework for understanding at which level change is required, and who has the authority to influence change. Using grounded theory methods, we identify approaches used by Nova Scotia past committee members to compensate for perceived shortcomings of the process. These include an undue reliance on other committee members, on the multidisciplinarity of the committee, and on past decisions. Using a policy analysis approach, we argue that centralization and systematization of the review process only partially address the shortcomings of the previous regionalized process. Lessons from Canada can inform policy discussions across all health systems, where similar challenges with the formulary review process have been identified. © 2016 The Authors. *The International Journal of Health Planning and Management* published by John Wiley & Sons Ltd.

## Introduction

Public reimbursement of drugs is a costly proposition for any health care system. On average, OECD countries spent $410 1$US Purchasing Power Parity (PPP), as reported by OECD (http://www.oecd‐ilibrary.org/content/datacollection/health‐data‐en) per capita on pharmaceuticals in 2013, ranging between $140 per capita in New Zealand to $633 per capita in Belgium. The total average expenditure on pharmaceuticals was $11 845 million. (OECD, 2016) Drug expenditure in Canada was at $34 425 million 2Using the PPP conversion factor of 1.1, as reported by World Bank (http://data.worldbank.org/indicator/PA.NUS.PPPC.RF) in 2015 (CIHI, 2015). Despite the absence of a nationalized drug insurance plan, approximately 37% of drugs in Canada are funded publicly, amounting to $12 598 million in 2015. Table 1 provides a detailed overview of drug expenditures across Canadian Provinces and Territories.

**Table 1:** Overview of health expenditures in Canadian Provinces/Territories in 2015 Millions ($ CAD)

Province/territory	Federal health transfer ‡	Total health expenditure (total)*	Total health expenditure (public)*	Drug expenditure (total)*	Drug expenditure (public)*	Population size (#) †	Public drug expenditure per capita	Public drug/total drug spending
Alberta	3980	29 260.1	21 705.3	3724.0	1419.9	4 216 875	336.72	0.38
British Columbia	4446	27 582.9	19 989.4	3374.4	1030.1	4 703 939	218.99	0.31
Manitoba	1227	8972.1	6862.0	1094.8	418.2	1 298 591	322.04	0.38
New Brunswick	716	4766.7	3376.8	786.9	220.3	754 164	292.11	0.28
Newfoundland	502	3685.8	2825.0	513.3	164.0	528 190	310.49	0.32
Nova Scotia	896	6197.2	4390.4	1026.2	303.5	945 121	321.12	0.30
North West Territories	42	606.9	525.8	30.5	17.4	44 253	393.19	0.57
Nunavut	35	521.5	487.1	24.1	13.7	37 026	370.01	0.57
Ontario	13 089	81 768.7	55 072.9	13 617.5	4866.9	13 850 090	351.40	0.36
Prince Edward Island	139	939.3	701.0	125.7	37.8	146 679	257.71	0.30
Quebec	7844	46 869.8	32 915.6	9067.9	3674.2	8 284 656	443.49	0.41
Saskatchewan	1070	7573.7	5828.9	1012.0	417.7	1 138 897	366.76	0.41
Yukon	35	399.7	320.3	27.7	14.5	37 288	388.87	0.52
**Canada**	**34 021.00**	**219 144.3**	**155 000.3**	**34 425.0**	**12 598.2**	**35 985 751**	**350.09**	0.37

Easily visible national totals or national averages.

* Canadian Institute for Health Information. National Health Expenditure Database. October 28,2015. www.cihi.ca

† Statistics Canada, CANSIM Tables, 2015 Estimates http://www.statcan.gc.ca/daily‐quotidien/151216/dq151216e‐cansim‐eng.htm

‡ Federal Support to Provinces. 2015/16 Budget Estimates. http://www.fin.gc.ca/fedprov/mtp‐eng.asp

Under the constraints of limited public resources, the decision to place a drug on a reimbursement list or the formulary is a key resource allocation issue. Given the critical importance of this decision, most of the OECD countries have a process for the review of drugs that allows for the decision to be rooted in evidence (Barnieh *et al.,* 2014). The majority of the OECD systems rely on multiple lines of evidence in the review process. Twenty‐two of the 34 countries, including Canada, require that economic evaluation be considered alongside other evidence. Only five of the countries met what Barnieh *et al.* (2014) refer to as “*the highest standards of transparency, requirement of evidence, and ability to appeal*”. Canada was assessed as meeting two of the criteria, transparency and requirement of evidence. Our study unpacks the Canadian process to show the degree to which these criteria have changed over time.

We assess the cancer *formulary process* in Canada and its evolution from a regionalized to a centralized review process, with a focus on the use of economic evaluation to support the review. The term *formulary process* is understood to mean the process of placing a drug on the public formulary, after it passes a basic safety and efficacy inspection by Canada's Federal Government. 3Health Canada, a Department of Canada's Federal Government issues a Notice of Compliance, after inspecting drugs for efficacy compared to a placebo, basic safety and product quality. The decision to place a drug on the formulary is made at the Provincial/Territorial level by the Minister of Health, therefore all review processes/expert committees are advisory. Cancer drugs are subject to a separate review process, largely because they are prohibitively expensive, yet widely needed. In Canada, more than 290 per 100 000 population were diagnosed with cancer in 2012, which was higher than global average (Cancer Research UK, 2014).

Since 2011, the formulary review in Canada is centralized. Recommendations of the pan Canadian Oncology Drug Review (pCODR) are available to all Canadian health ministries for consideration. 4Some other Canadian provinces, for example Ontario, continue to rely on supplementary provincial processes. Prior to the centralization, formulary reviews were conducted at the Provincial or Territorial level. For example, between 2006 and 2011, the province of Nova Scotia (NS) relied on the recommendations of the NS Cancer Systemic Therapy Policy Committee (NS Committee). We use this committee as an exemplar case study, because it was the first in Canada to both (i) focus on the review of cancer drugs exclusively; and (ii) formalize the incorporation of economic evidence into the review process.

Relying on past studies of formulary review committees/processes, and on qualitative interviews with the NS Committee, we identify the challenges experienced by committee members with the use of economic information in the multi‐criterion decision context. Challenges at the macro‐level included the low trust in economic evaluations because of pharmaceutical sponsorship and lack of regulation; and the absence of information related to available budgets. At the meso‐level, challenges included the variation of economic methodologies and models; the uncertainty associated with applicability of results to relevant contexts; the absence of relative criteria for the interpretation of economic results; and the lack of a consistent framework for the balancing of economic with other criteria. At the micro‐level, the challenges included the variation in economic training across individuals involved in the review, and the resultant undue reliance on the judgment of the economic expert(s).

A policy analysis entails the “breaking up of a policy problem into its component parts, understanding them and developing ideas about what to do.” (Patton *et al.,* 2013) A policy analysis evaluates policy options and their ability to respond to the problem components. The macro‐, meso‐, and micro level classification provides an analytical framework for the policy analysis of the formulary review process by organizing the relevant component parts. The expectation is that a central policy process is more readily able to respond to meso‐level challenges, with an influence over the macro‐level issues. The analysis of the centralization and standardization reveals, however, that issues remain with the lack of relevant reference criteria for the interpretation of economic studies, and with the insufficient guidance around the weighing of multiple decision criteria.

In section 2 of this paper, we describe the evolution of the cancer formulary review process in Canada. We focus on the recent centralization and systematization of the process, as well as on the guidelines and incorporation of economic evidence alongside other criteria. In section 3, we describe our methods. In section 4, we describe our findings of the challenges associated with a regionalized process. In section 5, we provide an analysis of the centralization and systematization as a policy response to the challenges of the regionalized process. Finally in section 6, we provide concluding remarks.

## Context

### The centralization and systematization of the Canadian cancer formulary review process

In Canada, intravenous cancer drugs are paid out of hospital budgets or through Provincial Cancer agencies. Oral drugs may be covered though provincial drug plans, which are available in some Provinces to select groups of patients; oral drugs may also be covered through one of six Federal drug programs (Drug Coverage, 2015). As there are at least 18 cancer drug plans in Canada, with distinct formularies. As a result, access to cancer drugs is not equitable for all Canadians. (Chafe *et al.,* 2011)

Decisions regarding the drug formulary are made at the jurisdictional level, typically by the Minister of Health, who relies on expert advice. Since 2011, expert advice is available through the centralized pan‐Canadian Oncology Drug Review (pCODR). Prior to 2011, most jurisdictions had relied on expert advisory committees, and some continue to rely on regionalized committees to date. Table 2 provides an overview of Provincial review committees that have existed prior to 2011, and also those that continue to operate after 2011. In addition, several other pan‐Canadian organizations support the pCODR process and are described in Table 3.

**Table 2:**
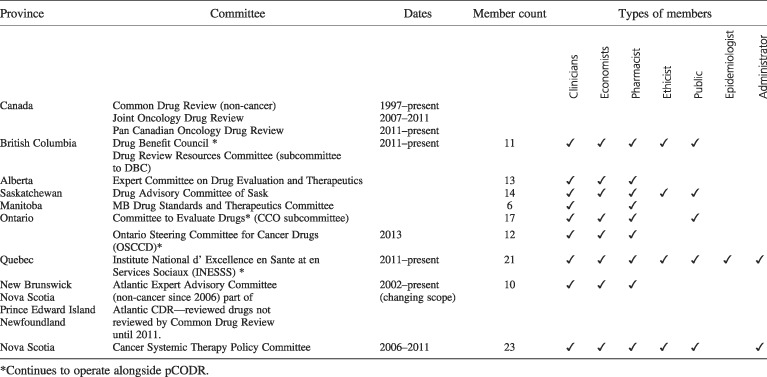
Provincial/territorial advisory committees

**Table 3:** Canadian organizations involved in the cancer drug review/funding process

Name	Mission/description	Role in formulary process
Canadian Cancer Society (CCS)	The CCS is a national, community‐based organization of volunteers whose mission is the eradication of cancer and the enhancement of the quality of life of people living with cancer. Read more: http://www.cancer.ca/en/about‐us/our‐mission/#ixzz49g3QIdZo	Indirect. Supporting documentation may be provided to support submissions to the pCODR process from patients or provincial advisory groups.
Canadian Association of Provincial Cancer Agencies (CAPCA)	CAPCA is an inter‐provincial organization of provincial/territorial cancer agencies/programs engaged in cancer control. CAPCA supports the reduction of the burden of cancer on Canadians and advocacy for cancer care and control. Collectively, the members of CAPCA work to reduce the burden of cancer by promoting the highest quality of care and services for all Canadians affected by cancer and at risk of cancer, and implementing the cancer control strategy in their respective provinces. http://www.capca.ca/	Specific member organizations are often the submitters of the patient perspective documents for purposes of the pCODR process.
Canadian Cancer Research Alliance (CCRA)	An alliance of organizations that collectively fund most of the cancer research conducted in Canada—research that will lead to better ways to prevent, diagnose, and treat cancer and improve survivor outcomes. Our members include federal research funding programs/agencies, provincial research agencies, provincial cancer care agencies, cancer charities, and other voluntary associations. http://www.ccra‐acrc.ca/index.php	Indirect. Member organizations may fund some clinical evidence brought to the pCODR process.
Canadian Partnership Against Cancer (CPAC)	The CPAC s an independent organization funded by the federal government to accelerate action on cancer control for all Canadians. The Partnership works with cancer experts, charitable organizations, government, cancer agencies, national health organizations, patients, survivors, and others to implement Canada's cancer control strategy. http://www.partnershipagainstcancer.ca/who‐we‐are/	Involved in some of the submissions from patient groups.
Council of Canadian Cancer Registries	The Canadian Cancer Registry is an administrative survey. Beginning with cases diagnosed in 1992, cancer incidence collected by provincial and territorial cancer registries have been reported to the Canadian Cancer Registry, which is maintained by Statistics Canada.	Indirect. Information may be used by Provincial Advisory Groups in their submissions for purposes of the pCODR process.
National Breast Cancer Research Framework	The National Breast Cancer Research Framework is the product of a broad‐based, comprehensive, and collaborative process. It reflects the input of funders, breast cancer survivors, researchers, and clinicians from across the country and, looking ahead, offers a coherent vision of the most promising areas for breast cancer research.	Indirect. May connect researchers with funding opportunities, which lead to evidence used in the pCOD process.
Pan‐Canadian Pharmaceutical Alliance (PCPA)	The PCPA was formed in 2010 under the Council of the Federation to join provinces and territories to negotiate prices for publicly covered drugs. The PCPA examines all drugs recommended for funding by the Common Drug Review and the pan‐Canadian Oncology Drug Review, then decides whether joint pan‐Canadian negotiations occur.	Post pCODR process. Price negotiations, if successful, may lead to a re‐assessment of a drug by pCODR.
Patented Medicine Prices Review Board	The PMPRB is an independent quasi‐judicial body established by Parliament in 1987 under the Patent Act. The PMPRB protects the interests of Canadian consumers by ensuring that the prices of patented medicines sold in Canada are not excessive. It does this by reviewing the prices that patentees charge for each individual patented drug product in Canadian markets. http://pmprb‐cepmb.gc.ca/home	Independent process. Potential overlap between drugs reviewed by PMPRB and pCODR.

The pCODR incorporates evidence/documentation in four domains: overall clinical benefit, cost‐effectiveness, alignment with patient values, and feasibility of adoption into the health system (pCODR deliberative framework). The review relies on published clinical trial results, economic studies submitted by the drug manufacturer, submissions from patient groups, and submissions from Provinces. The stages of the review are described below in a comparative context. The process results on one of three possible recommendations: fund, do not fund, or funding conditional (typically upon a reduction in the price of the drug). Recommendations are available publicly.

To highlight differences between the regionalized and centralized review process, we focus on an exemplar case study: the Nova Scotia Cancer Systemic Therapy Policy Committee (NS Committee). The NS Committee is not representative of all provinces, but did experience challenges similar to those encountered by other committees and described in the literature.

The NS committee was the first in Canada to focus exclusively on the review of cancer drugs, and also to formalize the incorporation of economic evidence into the review process. The NS Committee was phased out in 2011.

The NS process consisted of a review of clinical evidence and economic evaluation, and a joint committee discussion of clinical, economic, and ethical considerations. The clinical evidence was reviewed by the cancer site team prior to full committee consideration. The economic evidence was reviewed by a health economist. Members of the site team and the health economist were voting members of the NS committee (23 members). The committee met in person to deliberate. Each meeting consisted of a clinical presentation, an economic presentation by the health economist/committee member, and a deliberation. All committee members were asked to vote electronically within a two‐week period, and a simple majority vote was considered. The pCODR separates the review into two distinct phases. CE is reviewed by the Clinical Guidance Panel (CGP) and PE is reviewed by the Economic Guidance Panel (EGP). There is no overlap in membership between the CGP, the EGP, and the pCODR Expert Advisory Committee (pERC), who deliberates and votes on site via secret ballot (16 members). Both the CGP and the EGP have the opportunity to connect with the drug manufacturer to discuss the CE and/or PE.

**Figure 1:**
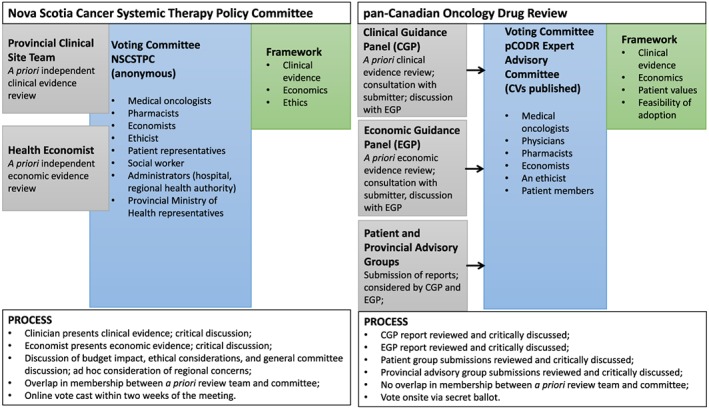
A regionalized versus a centralized review process. [Colour figure can be viewed at wileyonlinelibrary.com]

Figure 1 provides a visual representation of both processes (Nova Scotia Department of Health and Wellness, 2011; Kirby et al., 2008; Pan‐Canadian Oncology Drug Review, 2011) with the goal of highlighting the differences between them.

Both the NS Committee and the pCODR deliberative frameworks explicate that CE and PE, and their quality, be considered. The pCODR framework also includes patient values, 5There is not standard measure of “patient values”. Instead, the committee accepts submissions from patient groups. and adoption feasibility, including a consideration of the budget impact. In NS, the discussion of patient values fit under the category of “ethics”, which also included discussions of unmet need or the profile of the patient population. Neither framework explicates the approach to combining evidence, nor guides the interpretation of economic results by providing relative reference criteria, such as a cost‐effectiveness threshold or the size of the total budget.

Recommendations are made despite incomplete guidelines. We assess the approach taken by individual members of the NS Committee to (i) the use of multiple lines of evidence in absence of a weighing framework; and (ii) the interpretation of PE in absence of relative reference criteria; and (iii) approaches used to compensate for perceived shortcomings of the process. We discover challenges consistent with the literature, and additional challenges specific to the Canadian and/or Nova Scotia context.

## Methods

The goal of our study was to discuss the extent to which a centralization and systematization of the cancer formulary review responded to shortcomings identified in the regionalized process. To highlight the shortcomings of the regionalized process, we synthesize extant knowledge and supplement it with a case study. To assess the appropriateness of the centralization and systematization, we use a policy analysis approach.

### Literature

We conducted a narrative summary of the literature (Dixon‐Woods *et al.,* 2005; Kastner *et al.,* 2012) describing the challenges experienced by drug formulary committees. The narrative summary or narrative review is a relatively informal approach, the goal of which is to provide a reflexive and interpretive account of the extant knowledge. (Dixon‐Woods *et al.,* 2005). We retrieved 154 titles thought the Scopus database. We screened in two stages, first at the titles/abstracts level, and second at the full text level. Studies advanced, when they discussed drug reimbursement decisions in health systems of the OECD, focusing on policies and processes, and the use economic evaluation in a multiple criteria context. (See Table 4 for detailed search and selection strategy). Thirty‐seven studies were included in the narrative summary.

**Table 4:** Literature search and selection for a narrative review

Search syntax (SCOPUS)	Number of titles retrieved
( TITLE‐ABS‐KEY ( "drug reimbursement" ) AND TITLE‐ABS‐KEY ( decision ) )	122
( TITLE‐ABS‐KEY ( "drug fund*" ) AND TITLE‐ABS‐KEY ( decision ) )	34
Number of titles after duplicates removed	154
***Selection criteria***	
Inclusion	• Discusses the process of decision‐making regarding placing drugs on public formularies or reimbursement lists; • Discusses challenges encountered in the process; •Discusses facilitators of the process;
Exclusion	• Discusses the reimbursement of one particular drug; • Discusses policies around drug reimbursement that are not about the decision regarding reimbursement; • Focuses on countries outside the OECD; • Not related to drug reimbursement policies;

#### NS committee case study

#### Case study population and sample

The study population consisted of the members of the NS Committee. Twelve of the 23 members of the NS Committee (not all served on the committee at once) agreed to participate in the study, for a response rate of 52%. The response rate is relatively high in the context of studying health care professionals in a lengthy process, and including questions that could be perceived as sensitive (Abdulaziz *et al.,* 2015; Edwards *et al.,* 2009; James *et al.,* 2011; Nicholls *et al.,* 2011). Committee members were contacted after 2011, when their responses could no longer influence the operations of the committee or the committee members' own position. This increased the likelihood of receiving complete responses.

#### Case study—data collection

Data were collected in two phases, the first of which facilitated the second. The first phase consisted of participant observation between 2006 and 2011. DW was a member of the committee and served as a health economist. Observation revealed that PE was presented without necessary contextual information, and that committee members lacked confidence in PE. This facilitated the framing of the research problem and development of further research instruments. In the second phase, data were collected using two qualitative interview techniques: (i) open‐ended questions, and (ii) a discussion based on scenarios. Both were used in the same interview sequentially. Phase 2 data collection took place between January and May 2012.

Respondents were asked five open‐ended questions about their understanding of what PE is, what role it plays in the committee process, and how they use PE in their own deliberations. The questions were followed with a series of up to five scenarios simulating a situation where a drug has been submitted for approval to the provincial formulary. Each scenario included information about the clinical effect, cost‐utility ratios, quality of evidence, patient characteristics, and disease characteristics. Respondents were instructed to think through the scenario out loud. Interviews were taped, transcribed and identities were concealed.

#### Case study analysis

We use the grounded theory approach and associated coding techniques (Strauss and Corbin, 1990) to identify how committee members describe the process, the strengths and shortcomings of the process, and the ways in which they compensate for the perceived shortcomings. DW, LG, and NH coded interviews using open coding to establish common concepts and categories. DW, LG, and NH used axial coding to connect the categories. DW and LG used selective coding to develop the narrative. Strengths and shortcomings were grouped based on content and level of concern (micro, meso, and macro). Approaches used to compensate for perceived shortcomings were teased out using grounded theory. Scenarios were analyzed with the intent to identify approaches used to compensate for perceived shortcomings. Of particular interest were reactions to ambiguity, uncertainty and/or lack of sufficient information, the respondents' dilemmas and resolutions thereof.

#### Policy analysis

Policy analysis entails the “breaking up of a policy problem into its component parts, understanding them, and developing ideas about what to do.” It is a part of a larger policy planning process, which has a longer time horizon, uses more advanced methods, and includes implementation. (Patton *et al.,* 2013) Policy analysis is “an applied social science discipline which employs multiple methods of inquiry, in the contexts of argumentation and public debate, to create, critically assess, and communicate policy relevant information” (Dunn, 1994) [with the intent of] “finding solutions to practical problems” (Dunn, 2012). Policy analysis after problem identification and entails the assessment of merits and demerits of competing policy options. We use a macro‐, meso‐, micro‐level hierarchical framework to categorize the component parts of the policy problem. We propose that component parts in the higher levels of the hierarchy are more plausibly addressed via a centralized and systematized policy approach. We then critically assess the extent to which this proposition has been operationalized in the case of the Canadian cancer drug reimbursement process.

## Challeges of a Regionalized Review Process

### Literature

Among the health care systems of the Organization for Economic Cooperation and Development (OECD), all by one have at least one public drug formulary (Barnieh *et al.,* 2014). Comparisons of cancer formulary listings reveal inconsistencies across regions (Chafe *et al.,* 2011; Mihajlovic *et al.,* 2015; Chabot and Rocci, 2010; Cheema *et al.,* 2012). Studies that examine the use of PE in formulary processes in subsets of OECD systems focus on: (i) challenges inherent to the production and use of economic studies; and (ii) challenges of balancing economic criteria with clinical, ethical, feasibility, and other criteria.

The role of economic evaluation in the formulary decision process has changed over time. Earlier studies note the need to formally consider PE in decisions in Canada (Martin *et al.,* 2001; PausJenssen *et al.,* 2003; Duthie *et al.,* 1999) and elsewhere (Duthie *et al.,* 1999). Increasingly the use of PE has been built into the decision process (Barnieh *et al.,* 2014; Bryan and Williams, 2007), specifically in the context of cancer drugs (Yong *et al.,* 2013; Hoch *et al.,* 2012; Hoch and Sabharwal, 2013). Only eight of the 35 OECD systems neither require economic information nor have guidelines for its use. Most formulary processes involve committees, whose funding recommendations may or may not be binding (Barnieh *et al.,* 2014).

Challenges inherent to economic evaluations of drugs include methodological/technical, understandability, and issues of credibility. Studies can be difficult to understand and interpret by their users, because of insufficient economic training (PausJenssen *et al.,* 2003; Duthie *et al.,* 1999; Bryan *et al.,* 2007; Singer *et al.,* 2000). Decision makers prefer short summaries and/or research abstracts, and many do not feel equipped to use economic studies (Berry *et al.,* 2010; Thurston *et al.,* 2008; Hoffman *et al.,* 2002; Weizman *et al.,* 2008). Studies of the use of PE by formulary committees identify as problems the poor quality of studies, the uncertainty surrounding data used in economic estimations, the variation in perspectives taken, and insufficient sensitivity analyses (PausJenssen *et al.,* 2003; Yong *et al.,* 2013; Godfrey and Parrot, 2008). Committees appear to have a preference for their own crude analyses over formal evidence (PausJenssen *et al.,* 2003).

The credibility of studies is compromised when they are sponsored by the pharmaceutical industry (Bryan *et al.,* 2007; Berry *et al.,* 2010; Drummond *et al.,* 1997) and not regulated (Cookson and Hutton, 2003). Pharmaceutical estimates consistently overestimate cost‐effectiveness as compared to academic estimates (Bell *et al.,* 2006; Eddama and Coast, 2008; Miners *et al.,* 2005), and favorable PE is more readily published (Shemilt *et al.,* 2010).

In the context of the decision‐process, studies note that PE required to make decisions is often not available at the time that it is needed (Hasle‐Pham *et al.,* 2005; Bryan *et al.,* 2007; Hartz and John, 2009). The problem in part originates with available clinical data being insufficient to support economic evaluations (Godfrey and Parrot, 2008). In general, there appears a dissonance between the intended use of economic evaluations and their actual use. (Berry *et al.,* 2010; Thurston *et al.,* 2008; Lyles, 2001)

A second type of challenge with the use of PE is the need to embed it within other decision criteria and an unruly decision making context. Efficiency considerations often fall by the wayside and are trumped by evidence of clinical benefit, political and/or other considerations (Martin *et al.,* 2001; Eddama and Coast, 2008; Boon *et al.,* 2015; Armstrong *et al.,* 2010). There is no agreement on what these decision criteria should be, how important they should be relatively to one another, nor is there a common framework for how they should be used in combination (Chabot and Rocci, 2010; Boon *et al.,* 2015; Franken *et al.,* 2015; McDonald *et al.,* 2015; Franken *et al.,* 2014; Cleemput *et al.,* 2012; Drummond, 2013; Franken *et al.,* 2013). There is also a lack of guidance to the interpretation of evidence, including PE (Wranik, 2008; Franken *et al.,* 2012). Last, the decision‐making context is characterized by inflexible budgets, clustered decision making requirements, and timing requirements that are misaligned with the availability of evidence (Hasle‐Pham *et al.,* 2005; Bryan *et al.,* 2007; Singer *et al.,* 2000; Eddama and Coast, 2008; Hartz and John, 2009).

#### Case study results

This section presents the issues discussed by NS Committee members as they relate to the use of economic evidence among other criteria in the formulary process, the perceived shortcomings of the process, and the ways in which individuals compensated for those shortcomings. Building on the literature and using a content analysis, we classify the issues discussed into five themes, and at three levels. This emergent analytical framework facilitates the policy analysis (Table 4). Respondents discussed the nature of economic evidence: (i) the value of using economics in decision‐making; (ii) the challenges of using economics; and (iii) using economics in a multi‐criteria context. They also discussed policies and processes: (iv) the composition of the committee; and (v) the elements of the formulary process.

The three levels at which strengths and shortcomings are discussed are conceptualized as follows. The macro‐level is the broadest and relates to the conceptualization of a decision process, the relevant information to be considered, and the types of stakeholders to be involved. At this level, broad normative and/or system level issues are addressed. The meso‐level relates to the specifics of a particular decision process, such as the methods of incorporating information agreed upon at the macro‐level, or involving the stakeholders. The concerns are expressed from the perspective of the committee as a whole. The micro‐level relates to the operationalization of the decision process, such as the logistics of meetings and voting activities. The concerns are expressed from the perspective of the individual and their actions in the context of the committee.

The analytical framework is populated with results of the case study as described below (Table 5). 6We are not able to identify the respondents by role (e.g., clinician, economist, patient representative), as several roles were represented by only one person, and disclosure would violate confidentiality. Further quotations from NS Committee members are presented in Appendix 1.

**Table 5:** Analytical framework for policy analysis

Level issue	MACRO	MESO	MICRO
Value of using economics	Normatively, should we use economic evidence in health care resource allocation?	Should this particular committee use economic evidence in the cancer drug funding decisions in Nova Scotia?	Should individual committee members use economic evidence in their own vote?
Challenges with economics	What are the general methodological and practical challenges in the production of economic evaluations?	What are the challenges with the use of the economic evaluations by a Nova Scotia committee?	What are the challenges with the use of economic evaluations experienced by individual committee members?
Multi‐criteria decision context	Normatively, which types of evidence should be used and how?	Which types of evidence should this particular committee use and how?	Which types of evidence should the individual committee members use and how?
Composition of the committee	What are the normative arguments for and against the notion of a multidisciplinary committee as the body to recommend cancer drugs for public funding?	What are the benefits and drawbacks of the particular composition of the committee in Nova Scotia?	How do individual committee members respond to the multidisciplinary nature of the committee?
The decision process	In general, how should economic evidence be built into the decision process?	At the level of the Nova Scotia committee, how should economic evidence be built into the decision process?	How should each individual committee member use economic evidence to support the committee decision process?

### The value of using economics in decision making

Respondents were not unified on the fundamental normative question, whether economics should play a role in the formulary process. Most, but not all, believed in the importance of considering economic and financial criteria.

At the macro‐level, the perceived importance of economics ranged from being one of the most important factors to consider and a good tool for resource allocation, to being one piece of the equation on the one hand, and the least important factor.

“*…the economic factor, without a doubt, has to be one of the biggest things given today's environment….*”“*…economics is (…) probably the least important thing. It's the thing we wrestle with the most, but it's the least relevant in so many ways.*”

The formal inclusion of economics into the NS process was praised. Curiously, some respondents expressed concern that fellow committee members were not sufficiently committed to the use of economics, specifically clinicians on the committee. Some respondents indeed noted that their vote was based primarily on clinical considerations.

“*… my faith in cost‐effectiveness analysis is on a very low level, you know, I've always tended to put my interest in the clinical data.*”

Other respondents perceived their own reliance on economics as potentially controversial.

“*…because I am a bureaucrat (…) unlike some folks on this committee, I think the budget means a lot more to me….*”

And others suspected economics might be used to pursue political goals.

### Challenges with the use of economics

Respondents discussed challenges with the use and interpretation of economic studies. A macro‐level concern voiced by nearly every committee member was the production of economic reports. The financing of economic evaluations by the pharmaceutical industry, coupled with the lack of regulation surrounding economic evaluations, resulted in a mistrust in the results of those studies.

“*…pharma has a vested interest in presenting the information in the best light.*”

“*…the drug company (…) can manipulate the data, I mean, you can make anything look good.*”

Economic methodology was called into question, as it was seen by some to be speculative rather than definitive.

“*…the number itself is an estimate at best (…) I'm looking for validity (…).*”

“*…the formula for QALY, there is a subjective factor involved here…*”

Meso‐level concerns included the region‐specific or population‐specific nature of some economic studies, whose results may not be transferrable to the local context. For example, the use U.S. or British utility data was questioned in the Canadian context, or Ontario pricing data in the Nova Scotian context.

“*…a lot of the time they are not relative to the Canadian market, they are not reflecting Canadian prices and other health outcomes…*”

Respondents also noted the lack of consistency in economic studies: the types of models used, the data are used, and the way that they are reported. This problem is closely linked to the macro‐level lack of regulation, and the funding by pharmaceutical industry.

“*… some analyses appear to be of better quality than others….*”

“*…different for‐hire companies (..) do variable jobs, some do a very good job, and some frighteningly bad.*”

A central micro‐level challenge is the difficulty with the understanding of economic information. Committee members explicitly reported that they struggle with the understanding of the economic concepts, and in particular struggle with understanding the concept of cost‐utility. In addition, the responses to various questions reveal a lack of understanding of technical details. Many committee members did not use the economic terminology appropriately.

“*…(cost utility) is the term I have a hard time with….*”

“*…Cost utility I've never been absolutely on top of..*”

The lack of understanding can be viewed as the responsibility of individual committee members. In the context of a multi‐disciplinary committee, however, the problem reflects a systemic issue of sufficient economic training and improved knowledge translation.

### Using economics in the context of multi‐criteria decisions

Our narrow question about the most important elements of an economic report was interpreted more broadly; the types of evidence to be included in the formulary process were discussed. Respondents commented on the importance of including CE, PE, ethical decisions, patient care perspective, alternatives, feasibility, total impact on the budget, and the overall budget. The pieces of economics evidence discussed generally revolved around the concept of getting a good idea of what is the value for money.

“ *… overall benefits, overall costs, is this good value (…) from the patient care perspective (…) from the overall public purse …* ”

“*…the CE, the PE (…) other dimensions (…) ethical and feasibility..*”

Respondents did not discuss a desire to include additional decision criteria (see Figure 1). A clearly noted gap, however, was the absence of information on a relative criterion that would facilitate the interpretation of the PE. Many commented on the lack of budgetary information.

“*… in this particular decision process, we never see the budget. We actually have no idea (…) I'd rather if there is a budget…*”

“*We don't usually go into meetings saying this is the budget that you have available.*”

And one commended on the apparent difference between the intended and actual use of economic information.

“*The idea is that you have a ranking of your programs (by ICER) (…) and as you slot a new more efficient program in, a less efficient program maybe drops out of the funding budget (…) that's not how the healthcare system is run….*”

Nearly all committee members indicated a strong need for a consistent framework as key to the use of multiple lines of evidence. We expand on this under “The decision process”.

### Composition of the committee

The normative position of committee members was that the committee should be composed of multiple disciplines to create a balance of perspectives, as the understanding of various pieces of evidence varies by discipline. Furthermore, individual members assign a greater importance to specific types of evidence (e.g., clinical, economic, patient care), and as a group, the weights assigned are balanced.

Respondents discussed the advantages of having the particular mix of skills on the committee. One indicated there was a high number of clinicians, perhaps creating an imbalance and undermining other voices.

“ *The committee is quite unique. Everybody comes to the table with a different level of understanding (…) some people put more importance on some of the other things than the PE (…) people are weighing differently …*”

Respondents thought that they benefitted from the multidisciplinary nature of the committee, as they were able to discuss the evidence from various perspectives and were able to draw on the expertise of others when needed.

The difficulties with understanding the PE led many committee members to heavily rely on the interpretation of PE as presented by the health economist, DW, during the meetings.

“*Well for me, because it's so hard to understand the language, so I really look to the presenter to inform me, you know, of basically all the key information (…)* ”

“*… it's useful to have somebody like (health economist) and there is one other member of our panel who has a fair amount of economic background (…) because left on your own, without having an economics background, it's complex to understand …*”

The availability of the health economist is advantage in giving the opportunity to consult an expert. The disadvantage is that the approach results in several perspectives on the same interpretation of the economic information, rather than several interpretations. A subtle distinction that allocates disproportionate influence over the committee's decision to one member and dilutes the value of the multidisciplinary nature of the committee.

### The decision process

The decision process refers to the logistics of the committee reviewing and using evidence. In the broadest macro‐level sense, the process includes the production of all information and the training offered to it users. In the micro‐level sense it includes details of individual meetings.

A prominent meso‐level concern discussed by most respondents was the need for a consistent framework. Committee members felt overwhelmed by the amount and variety of evidence, and did not know how to optimize the use of multiple decision criteria. While a framework was in place to identify the broad types of information to consider, no guidance was provided as to the interpretation of detail or the relative importance.

“*We need to put a number and put a weight on these things to arrive at a fair but more importantly a legitimate and justifiable outcome (…) we're just looking at disparate pieces of evidence without putting it into that framework. And I choose to put one set of weights on this information, and you choose to put one set of weights on that information, to me, we're not arriving at a fair outcome.* ”

“*I want to make sure that if I actually make that recommendation, I have used the same decision points that I have used in every other decision or recommendation.*”

“*People just sort of make mental shortcuts (…) humans are naturally lazy; give me the least amount of information to make a decision (…) if it's cheap, it must be efficient …*”

Respondents valued the opportunity for multidisciplinary discussion and had ideas for its improvement.

“*…the dialogue for me is key (…) people going back and forth on things, that's where I really have to listen and try to figure out where I stand…*”

“*… just a five minute re‐cap at the beginning of the definitions of what those different terms mean might be helpful …*”

A number of committee members also commented on their sustained confusion, even after meetings and discussions.

“*Clinical benefit of a particular treatment (…) then the figures are thrown at me and they make some sense in terms of costing (…) you think, where does it end, or what do I take home from this? (…) too much information.*”

“*…a few times you leave and you just don't know how to vote, because you still don't get everything…*”

Additional suggestions included a longer‐term follow up to understand the implications of recommendations on patients and diseases.

To sum up, the normative position of the NS Committee members was that PE ought to be considered in a multiple criteria context, and by a multidisciplinary group. The practice of such consideration was obstructed by perceptions and skills of committee members. They questioned the credibility of economic reports on two accounts: (i) studies were funded by the pharmaceutical industry; and (ii) economic methods and assumptions appeared too abstract. Committee members stated and demonstrated that their understanding of economic concepts is incomplete and that the balancing of PE with CE and other evidence is challenging. Some experienced a sustained apprehension even after meetings and discussions.

NS committee members compensated for the perceived shortcomings of the process (Table 6) in a number of ways. Reliance on the interpretation of evidence by others was frequently discussed. Arguably one goal of a multi‐disciplinary committee structure, the reliance was seen as imbalanced. The health economists' critical assessment and interpretation of economic studies, for example, were taken at face value. More importance was assigned to clinical studies than might have been, had it not been for the lack of trust in economic evaluations. Committee members discussed relying on their own implicit weights in the use of multiple criteria, and their own implicit thresholds in the interpretation of cost‐effectiveness ratios. Most recognized that the budget was not properly considered.

**Table 6:** Framework results—issues discussed by committee members

Level issue	MACRO	MESO	MICRO
Value of using economics	• Economic information is generally important, as there is a need to carefully allocate resources. Not all committee members shared this opinion.	• The consideration of economic evidence is key to the committee's decision process. • Concerns about other committee members not using economics. • Concerns that economics is sometimes used to support Provincial political interests.	• Individual committee members felt it important that the economic evidence be considered, but felt ill equipped to meaningfully use it themselves.
Challenges with economics	• Practical challenges include the funders of economic reports and subsequent lack of trust in the results, and lack of regulation of the production of economic reports. • Methodology was seen as speculative rather than definitive.	• Concerns that studies were not applicable to local context, because of geography, and because of population differences from those used in the utility valuation. • Concern about the lack of consistency between economic studies, and a lack of clarity.	• Individual committee members found it challenging to understand economic terminology and methods, particularly that of cost‐utility. Terminology and concepts were conflated.
Multi‐criteria decision context	• The initial question of what an economic report should include was interpreted more broadly. Important to include clinical evidence, economic evidence, ethical aspects, patient care perspective, alternatives, feasibility, total impact on the budget, and the overall budget.	• A clear distinction was not made between a general and a NS committee specific evidence package. • The lack of a consistent framework, and the lack of important information, and specifically budgetary information were identified.	• Status quo not questioned. Discussion of the pieces of economic evidence focused on what was presented to the committee. • The lack of budget information was mentioned by several committee members.
Composition of the committee	• In principle, committees should be composed of multiple disciplines to create a balance of perspectives, as the importance assigned to various pieces of evidence varies between disciplines.	• The composition of the particular NS committee was not questioned. There was little discussion of needing more or fewer representatives from any particular discipline.	• Some concerns were raised about being able to have a voice in the presence of a number of clinicians or economists. • Some committee members felt unheard.
The decision process	• The importance of education of stakeholders was highlighted, particularly in economics. • The literature had highlighted the need for regulation of the production of economic reports. • Committee members would have liked long‐term follow‐up to understand the broader implications of their decisions.	• The need for a framework was highlighted. • The opportunity for multidisciplinary discussion prior to voting was appreciated. • Committee members stressed their reliance on the economic expert to interpret economic studies for them.	• There was a desire to have more time to discuss each drug, and more time to review the summary of evidence. • Some experienced a sustained confusion. Not sure how to vote after the meeting and discussion. Took mental shortcuts.

#### Policy analysis of the standardization and systematization of the review process

The classification of shortcomings of a process into the macro‐, meso‐, and micro‐level framework reveals that a centralization and systematization of the review process could feasibly address many of the challenges at the meso level. Macro‐level challenges, such as the sponsorship of cost‐effectiveness studies by the pharmaceutical manufacturer, the absence of a regulatory approach to economic evaluation, and the lack of training in economics available to clinicians, decision makers, and administrators do not fall under the scope of a national review process such as pCODR.

A centralization and systematization of the review process would conceivably have the most influence over meso‐level challenges. Most NS Committee members explicitly stated or implied a need for a more functional framework to (i) decrease the confusion around the use of multiple criteria; and (ii) increase consistency between drug reviews. The lack of a budget was noted as problematic, as was the lack of a cost‐effectiveness threshold. These observations are consistent with the literature, which calls for improved frameworks (Cleemput *et al.,* 2012; Drummond, 2013; Franken *et al.,* 2013; Dionne *et al.,* 2015) and/or information on relative criteria, such as a threshold (Franken *et al.,* 2012) or budget amounts (Bryan *et al.,* 2007; Eddama and Coast, 2008).

We identified ways in which committee members compensated for the perceived shortcomings of the process (Table 7). Many noted a heavy reliance on the health economist to interpret PE, and a belief that fellow committee members are in a better position to interpret it (with less confusion). This is a sort of diffusion of social responsibility—where the group believes an action is important, but relies on others in the group to take the action (Darely and Latane, 1968). In addition, past decisions were used to assist with the interpretation of economic results, which is akin to creating an implicit cost‐effectiveness threshold. The multidisciplinarity of the committee was thought to compensate for the absence of clear weights, despite a concern that some committee members have more dominant voices. These solutions are sub‐optimal and require a systematization.

**Table 7:** Actions to compensate for perceived shortcomings

Shortcomings	Compensating actions
Lack of understanding of economic studies	• Reliance on health economist's interpretation; • Diffusion of responsibility—expectation that other committee members will use economics properly;
Mistrust in economic studies because of sponsorship and because of uncertainties;	• More importance assigned to clinical studies than otherwise would be;
Lack of weighing framework to guide multi‐criteria decisions;	• Perception that multidisciplinarity of committee compensates; • Creation of own implicit weights; • Reliance on others' interpretation;
Lack of a cost‐effectiveness threshold;	• Creation of own implicit threshold using comparison to past decisions;
Lack of information about the size of the total budget available for cancer drugs	• Budget not properly considered;

The centralization and systematization of the formulary process in Canada have only partially addressed the meso‐level challenged of the regionalized process. The pCODR process introduces several levels of review, including an *a priori* evaluation by the EGP and/or CGP, in consultation with the manufacturer and each other, and a deliberation by the pERC, a committee that includes at least two health economists, 7“At least two”, because two members sit in the role of health economist, while some members who serve a different role (e.g., clinical expert or patient representative) have had training in health economics. who do not serve on the EGP. The manufacturer is required to provide supplementary evidence and explanation during the review process, as well as the full economic model that can be re‐estimated by the EGP (Hoch and Sabharwal, 2013). This increases the manufacturer's accountability and stands to reduce the mistrust in the PE submission (Drummond, 2013). Furthermore, the pCODR process does not create an undue reliance on one individual to interpret economic evidence. The diffusion of social responsibility is reduced, since a larger proportion of individuals who participate in the pCODR process have training in economics.

The pCODR process retains three substantive shortcomings, however, and does not provide a sufficient guidance for the interpretation of evidence. Based on publicly available pCODR documents, the following challenges remain: (i) a lack of relative weights to assign to decision criteria; (ii) a lack of a cost‐effectiveness threshold; and (iii) no information about the size of the available budget. A review of review processes in 34 OECD systems reveals that only three specify a cost‐effectiveness threshold. (Barnieh *et al.,* 2014) The review does not assess the use of a weighing framework, or the availability of budget information.

The implementation of a weighing framework, a cost‐effectiveness threshold, and disclosure of the size of the available budget would support the consistency and transparency of reviews and recommendations. These are difficult to follow. Information about the size of the available budget is the responsibility of the government, and is external to the review process itself. Collection of this information would require a whole system change, at least in Canada, where the budgeting approach is fragmented, and budgets appear to have soft implicit caps. The specification of a weighing framework and a cost‐effectiveness threshold should be explicitly incorporated into the review process—the challenge lies with finding the appropriate values. A weighing scheme or threshold would be justifiable, if based on the preferences of decision makers or the patient‐public, which have been transparently measured. Such measurement should be conducted in all jurisdictions that aim to have a fair and transparent formulary process.

## Acknowledgement

This study was funded by the Canadian Cancer Society Research Institute (CCSRI) via the Canadian Centre for Applied Research in Cancer Control (ARCC) and by the Canadian Institutes for Health Research (CIHR).

This study was reviewed and approved by: Dalhousie University Research Ethics Board.

## Conclusions

In this study we argue that a centralization and systematization of a drug formulary process, as has taken place in Canada, have the capacity to address most meso‐level challenges with the use of economic evidence in a multi‐criterion decision context. Relying on the literature and on a case study of a pioneering Canadian regional committee, we categorize these challenges into an analytical framework that facilitates the critical assessment of policy changes.

The centralization and systematization of the Canadian process have not fully addressed the concerns with an absence of a weighing framework for the balancing of economic, clinical, and other criteria. These changes have also not developed a reference criterion for the interpretation of cost‐effectiveness studies, neither in the form of a threshold, nor in the form of budgetary information. The absence of firm guidelines is not uncommon in the OECD countries. Nonetheless, while the multidisciplinarity of a committee may conceivably compensate for a lack of a weighing framework, it remains problematic how economic studies can be properly interpreted in the absence of reference criteria.

## Appendix

**Table A1:** The value of economics in decision making

**Level**	**Quotations**
Macro	“*There is a fixed pool of resources out there. We have to allocate it is some way that hopefully has the most benefit to society. And economic evaluation is […] the tool that we use to allocate those resources between competing programs*”
	“*… have to use the funds that we have […] in the best possible way to benefit the greater good…*”
	“*…looking at the economics […] is one piece of the equation in terms of looking at the total* *picture on whether or not something should be recommended for funding.*”
	“*The economic factor, without a doubt, has to be one of the biggest things given today's environment …*”
	“*Economics is […] probably the least important thing. It's the thing we wrestle with the most, but it's the least relevant in so many ways.*”
Meso	“*Most of them (committee members) are oncologists or pharmacologists […] don't think with the dollar sigh hat on …*”
	“ *… in the finance world (this respondent self‐identified as belonging to this world) they really look at budget numbers […] in terms of the public purse and trying to make good decisions […] that's the thing we need to look at …*”
	“*… because I am a bureaucrat […] unlike some other folks on this committee, I think the budget means a lot more to me […] have to be able to explain, using pharmacoeconomic information, why I would support spending $10 million on a very few number of people …*”
	“*… we have to have a really good defendable process that incorporates the economics in it that can be understood by some politicians […] and the public*”
	“*People generally aren't angry when you approve a program, they're more angry when you don't approve the program.*”
Micro	“*My experience has been that it's generally secondary to clinical evidence. If there is strong clinical evidence that this drug extends life, it would have to be a pretty awful CE ratio on the other side to get them not to fund that drug.*”
	“*I am purely simply a clinician, so I mean it really goes back to […] the clinical benefit of a particular treatment.*”
	“*… there's been a few things that have been winners […] then you worry about what the economics is going to look like […] everything is basically hinged on the economics.*”
	“*My faith in the cost‐effectiveness analysis is really on a very low level, you know, I've always tended to put my interest in the clinical data.*”
	“*… it's useful to have somebody like [the health economist] and there is one other member of our panel who has a fair amount of economic background … because left on your own, without having an economics background, it's complex to understand …*”
	“*I don't go back and actually read the studies myself, so I take that information […] based on her [the health economist's] expertise …*”

**Table A2:** Challenges with the use of economics

**Level**	**Quotations**
Macro	*Funding conflict of interest*
	“*… you question the motives behind these studies. […] drug companies do these studies […] a lot of the studies aren't sound and they're not without bias*”
	“*… pharma has a vested interest in presenting the information in the best light.*”
	“*…if the report was written by the company who stands to gain from a decision to adopt their drug […] you have to accept an inherent bias in that.*”
	“*… the drug company […] can manipulate the data to make, I mean you can make anything look good.*”
	“*Some drug companies are going: Well, we know what it takes to get this drug approved so we're going to make sure the cost‐effectiveness is going to come in below what is reasonable.*”
	“*… the most important problem we face that most of the economic analyses are performed themselves by the companies interested in submitting and approving the drug or a group contracted by that particular company … so there is a conflict of interest.*”
	*Modeling*
	“*…pharmacoeconomic analysis is […] extrapolated […] makes it challenging […] the model is called into question […] the weaknesses of the information are presented …*”
	“*The number itself is an estimate at best […] I'm looking for validity […] basically whether I'm confident in that number*”
	“*…the formula for QALY, there is a subjective factor involved here …*”
	“*… a lot of the economics […] is based on a modeling system, and the systems make a lot of assumptions, and the assumptions are not always to my way of thinking appropriate…*”
Meso	*Lack of transferrability*
	“*… most of the economic reports are manufacture driven and they are not very good. A lot of the time they are not relative to the Canadian market or they're not reflecting Canadian prices and other health outcomes…*”
	“*…It's a little too abstract for my thinking, it's all based on a model […] you can poke holes in any of it”—a respondent discussing 100 healthy adults in Toronto, and using a ten year horizon when patients do not live this long.*
	“*…using comparators that are not close to what we would use in clinical practice. So this new treatment is better than that old treatment, but we discarded that old treatment five years ago with something else better.*”
	*Inconsistencies*
	“*…some analyses appear to be of better quality than others…*”
	“*…different for‐hire companies […] do variable jobs, some do a very good job, and some frighteningly bad.*”
Micro	*Lack of understanding*
	“*…it's really hard for me to understand but there is different utilities that you look at in terms of understanding benefits, I guess. I'm not sure exactly how to define utility.*”
	“*(cost utility) is the term I have a hard time with […] that cost utility piece is a difficult concept for me to understand.*”
	“*Cost utility […] fairly similar to cost‐effectiveness, but I am kind of drawing a bit of a blank here*”
	“*(cost utility) … this is where I start to break down a little bit […] putting a scientific value on the benefit of this drug compared to the cost of this drug …*”
	“*I am more of a science‐oriented person […] not saying this isn't scientific data but it's just to me it's too complex …*”
	“*Cost utility … don't quite know how to fit that in … not a term I consider very often.*”
	“*Cost utility, I've never been absolutely on top of.*”
	“*I get benefit and utility mixed up*”

**Table A3:** Using economics in the context of multi‐criteria decisions

**Level**	**Quotations**
Macro	“*… the clinical, the economics, the total impact on the budget, and then the ethics framework. So I think all the right pieces are there.*”
	“*… economic evaluation results […] size of the clinical results and the meaningfulness of them […] population we're treating […] I am biased toward first line treatments […] probably favour the (younger population)…*”
	“*… how much weight I should put on the economic analysis […] what is the cost per QALY […] are there any other options […]*”
	“*… the clinical evidence, the economic evidence […] other dimensions […] ethical and feasibility …*”
	“*… overall benefit, overall cost, is this good value […] from patient care perspective […] from overall public purse […] ethical decisions …*”
Meso	*Need consistent framework*
	“*I am a true believer in the power of economic evaluation and explicit decision making. We need to put a number and put a weight on these things to arrive at a fair but more importantly a legitimate and justifiable outcome … we're just looking at disparate pieces of evidence without putting it into that framework. And I choose to put one set of weights on this information, and you choose to put one set of weights on that information, to me, we're not arriving at a fair outcome.*”
	“*A sort of reproducibility and justifiability and legitimacy, all those things come out of having an explicit decision‐making process as opposed to just a committee of people that meet and make a decision on the basis that no one can quite quantify.*”
	“*…has to be almost pre‐determined before you can discuss the economics, you have to decide what the parameters are, as we often do it backwards…*”
	*Lack of budget*
	“*… we never really have a budget, we just have the impact on the budget. We never say, well, we only have $50 million to spend on chemotherapy … no overall budget for the program*”
	“*… in this particular decision process, we never see the budget. We actually have no idea. […] I'd rather, if there is a budget […] tell us what it is, give us the tools to work with so we can make our recommendations.*”
	“*If the budget was defined in black and while, that would be fine … but … in provincial governments and provincial budgets, there are many influences that make that budget very flexible.*”
	“*We don't usually go into the meetings saying this is the budget you have available.*”
	“*….there is a budget for oncology drugs in Nova Scotia … I have never talked to the minister about it, but I'm sure there is … at one point I talked to the Deputy about giving us the budget …*”
	“*… you have to look at the whole budget, and then you have to realize the decisions that you are making have to fall within the budget.*”
Micro	“*To me, I can't separate out like do I give this 25% weight and adopt it? […] I look at the total picture […] life years gained and the QALYs are just a component […] I also have to consider the strength of the whole analysis […] how much weight you put on those markers is really informed by the whole presentation …*”
	“*Well, for me because it's so hard to understand the language, so I really look to the presenter to inform me, you know, of basically all the key information […] I trust that she is presenting the information I need to make decisions because I have a difficult time deciphering.*”
	“*I don't have any budget in there with regards to the economic component so that's also a big piece that is missing …*”
	“*The budget often gets misused […] it's a small budget, so we don't care what the evaluation side says, we're just going to go ahead with it…*”

**Table A4:** Composition of the committee

**Level**	**Quotations**
Macro	“*… different professions and different disciplines view the exact same set of numbers and draw entirely different conclusions from them …*”
	“*[committee] brings together a good mix of people in term so their background …*”
	“*…one of the values of the committee is that there is various different perspectives …*”
	“*… there are so many clinicians […] so much clinical experience relative to economic experience on the committees …*”
	“*… it's a breath of fresh air to sit in that committee and have people legitimately debate the goodness and the appropriateness of a decision, that's a rare event […] (follows discussion of disconnect between physicians, administrators, industry interests)*”
Meso	“*I think our committee was actually quite effective […] we has some growing pains in the front end […] getting used to one another […] pick up on people's biases very quickly …*”
	“*Asked about committee: I think it's a fair process, it's been consistent across different evaluations we've done, and I don't think it could be done any differently given the information we were provided with.*”
	“*It's helpful to have an economic expert actually present the information, so that you can actually have a clearer understanding of what those terms mean.*”
	“*… having somebody interpret the data that is not vested with the actual industry is helpful …*”
	“*… it's useful to have somebody like [health economist] and there is one other member of our panel who has a fair amount of economic background […] because left on your own, without having an economics background, it's complex to understand …*”
	“*That's why we rely on people like (the health economist) and some of our national review committees, on the health economists there, to let us know does this make sense?*”
Micro	“*… there are so many clinicians […] so much clinical experience relative to economic experience on the committees …*”
	“*I'm not skilled like the other committee members.*”

**Table A5:** The decision process

**Level**	**Quotations**
Macro	“*… we need to tell them at an early stage: here is the economics, here is how and why you can trust it to include the clinical outcomes that you're most interested in […] if you don't have a clinically significant, clinically beneficial program, it's not going to be cost‐effective […] introduce residents to a little bit of economics.*”
Meso	*Discussion*
	“*…the committee themselves take the job extremely seriously […] they are a very very objective bunch of people around the room.*”
	“*…people are thinking about what they are doing […] not just reacting, not just following the flow […] not too many other processes allow that.*”
	“*The downside that has always troubled me […] we being human beings and not machines are influenced by how things are presented.*”
	*Change over time*
	“*I believe that over time, she has been able to understand what is the information that we need to hear […] she has done a better job in terms of being able to answer questions…*”
	“*Our economic evaluations in recent years have been presented consistently with a consistent format and flow and model […] very helpful.*”
	*Need for a framework*
	“*I want to make sure that if I actually make that recommendation, I have used the same decision points that I have used in every other decision or recommendation.*”
	“*… our specific decision in that committee is to maintain a relatively rigid process in terms of what we look at and to control as much of it as possible..*”
	“*People just sort of make mental shortcuts […] humans are naturally lazy; give me the least amount of information to make a decision […] if it's cheap, it must be efficient …*”
	“*I think I weight it the same […] if you see benefits of a drug helping people to live a lot longer, it certainly weighs on you […] But then ethically […] who am I to judge 3 months versus a year versus five years? […] I struggle with all that from a social perspective.*”
	“*To me, I can't separate out like do I give this 25% weight and adopt it? […] I look at the total picture […] life years gained and the QALYs are just a component […] I also have to consider the strength of the whole analysis […] how much weight you put on those markers is really informed by the whole presentation …*”
	*Reliance on the economic expert*
	“*It's helpful to have an economic expert actually present the information, so that you can actually have a clearer understanding of what those terms mean.*”
	“*… having somebody interpret the data that is not vested with the actual industry is helpful …*”
	“*… it's useful to have somebody like [health economist] and there is one other member of our panel who has a fair amount of economic background […] because left on your own, without having an economics background, it's complex to understand …*”
	“*I don't go back and actually read the studies myself, so I take that information […] based on her expertise …*”
	“*That's why we rely on people like (the health economist) and some of our national review committees, on the health economists there, to let us know does this make sense?*”
	*Need for long‐term follow‐up*
	“*… I pay a lot of attention to, now that we have some decisions under our belt historically […] how does this compare to something that we looked at last year? What decisions were made then?*”
Micro	“*… sometimes the meetings are not long enough […] the dialogue is cut off because they're trying to cram too many drugs into a meeting*”
	“*What I don't find helpful […] a long discussion on how we use five hear horizons and 20 year horizons, and some of the more technical details that have driven the outcome.*”
	“*…needs to be more time, but overall, I like the structure of the meeting…*”
	“*… just a five minute re‐cap at the beginning of the definitions of what those different terms mean might be helpful …*”
	“*executive summary […] would be helpful ahead of time*”
	“*… a few times you leave and you just don't know how to vote, because you still don't get everything …*”
	“*It's important to have it delivered in an understandable way […] people who present obviously try their best … I find myself sometimes I have no idea what they're talking about*”
	“*This is a debate I always stuggle with [examples of criteria] it's all those complicated issues in the evaluation […] and sometimes it becomes philosophical or the ethical debate slips into things.*”
	“*Clinical benefit of a particular treatment […] then the figures are thrown at me and they make some sense in terms of costing […] you think, where does it end, or what do I take home from this? […] too much information.*”
